# Supplementary material for: Causal Relationship between Aging and Anorexia Nervosa: A White-Matter-Microstructure-Mediated Mendelian Randomization Analysis
Source: Biomedicines. 2024 Aug 16;12(8):1874. doi: 10.3390/biomedicines12081874 (PMC11351342; doi:10.3390/biomedicines12081874)
Supplement: Supplementary file 1 [file biomedicines-12-01874-s001.zip › Supplementary Figure/Figure S4/Singe SNP plot of MR analysis for TL to ubm-b-1968.pdf]

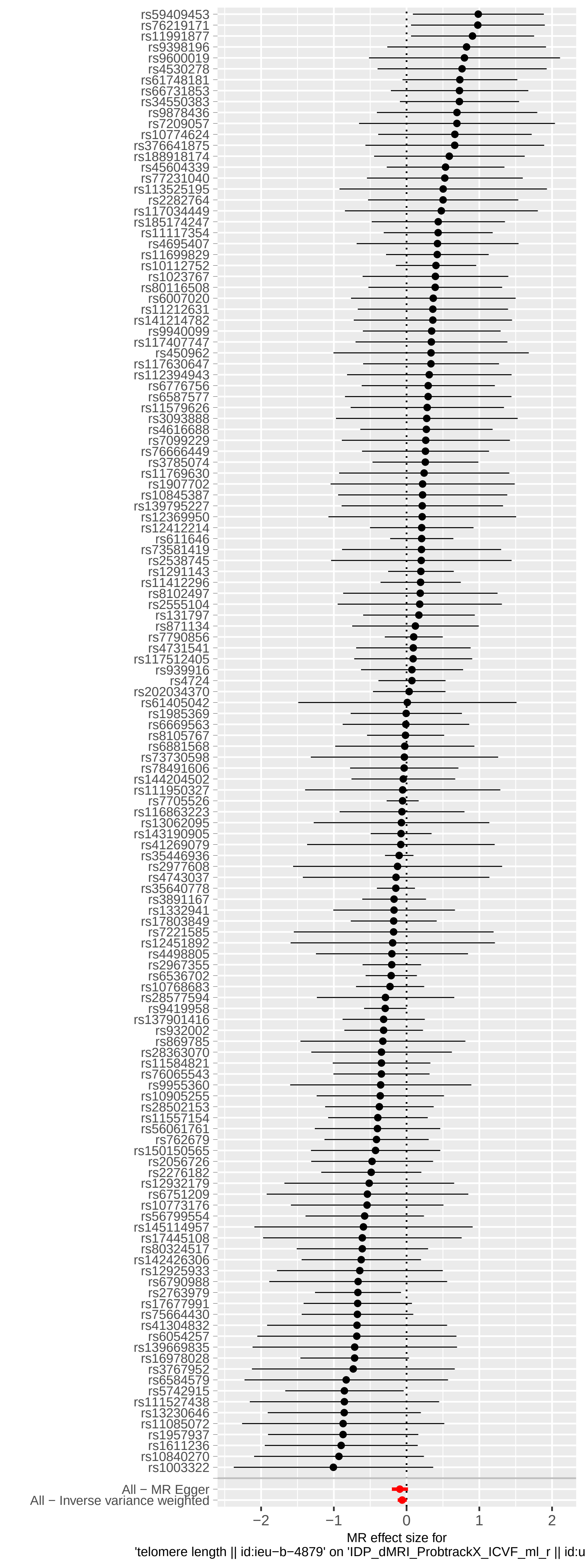
rs59409453
rs76219171
rs11991877
rs9398196
rs9600019
rs4530278
rs61748181
rs66731853
rs34550383
rs9878436
rs7209057
rs10774624
rs376641875
rs188918174
rs45604339
rs77231040
rs113525195
rs2282764
rs117034449
rs185174247
rs11117354
rs4695407
rs11699829
rs10112752
rs1023767
rs80116508
rs6007020
rs11212631
rs141214782
rs9940099
rs117407747
rs450962
rs117630647
rs112394943
rs6776756
rs6587577
rs11579626
rs3093888
rs4616688
rs7099229
rs76666449
rs3785074
rs11769630
rs1907702
rs10845387
rs139795227
rs12369950
rs12412214
rs611646
rs73581419
rs2538745
rs1291143
rs11412296
rs8102497
rs2555104
rs131797
rs871134
rs7790856
rs4731541
rs117512405
rs939916
rs4724
rs202034370
rs61405042
rs1985369
rs6669563
rs8105767
rs6881568
rs73730598
rs78491606
rs144204502
rs111950327
rs7705526
rs116863223
rs13062095
rs143190905
rs41269079
rs35446936
rs2977608
rs4743037
rs35640778
rs3891167
rs1332941
rs17803849
rs7221585
rs12451892
rs4498805
rs2967355
rs6536702
rs10768683
rs28577594
rs9419958
rs137901416
rs932002
rs869785
rs28363070
rs11584821
rs76065543
rs9955360
rs10905255
rs28502153
rs11557154
rs56061761
rs762679
rs150150565
rs2056726
rs2276182
rs12932179
rs6751209
rs10773176
rs56799554
rs145114957
rs17445108
rs80324517
rs142426306
rs12925933
rs6790988
rs2763979
rs17677991
rs75664430
rs41304832
rs6054257
rs139669835
rs16978028
rs3767952
rs6584579
rs5742915
rs111527438
rs13230646
rs11085072
rs1957937
rs1611236
rs10840270
rs1003322
All − MR Egger
All − Inverse variance weighted
−2
−1
0
1
2
MR effect size for
'telomere length || id:ieu−b−4879' on 'IDP_dMRI_ProbtrackX_ICVF_ml_r || id:u
